# The development and ex vivo evaluation of a computer-aided quality control system for Barrett’s esophagus endoscopy

**DOI:** 10.1055/a-2537-3510

**Published:** 2025-03-06

**Authors:** Martijn R. Jong, Tim J. M. Jaspers, Rixta A. H. van Eijck van Heslinga, Jelmer B. Jukema, Carolus H. J. Kusters, Tim G. W. Boers, Roos E. Pouw, Lucas C. Duits, Peter H. N. de With, Fons van der Sommen, Albert Jeroen de Groof, Jacques J. G. H. M. Bergman, on behalf of the BONS-AI Consortium

**Affiliations:** 1 Department of Gastroenterology and Hepatology, Amsterdam Gastroenterology, Endocrinology and Metabolism, Amsterdam UMC, University of Amsterdam, Amsterdam, the Netherlands; 2 Department of Electrical Engineering, Eindhoven University of Technology, Eindhoven, the Netherlands

## Abstract

**Background** Timely detection of neoplasia in Barrett’s esophagus (BE) remains challenging. While computer-aided detection (CADe) systems have been developed to assist endoscopists, their effectiveness depends heavily on the quality of the endoscopic procedure. This study introduces a novel computer-aided quality (CAQ) system for BE, evaluating its stand-alone performance and integration with a CADe system.

**Method** The CAQ system was developed using 7,463 images from 359 BE patients. It assesses objective quality parameters (e. g., blurriness, illumination) and subjective parameters (mucosal cleanliness, esophageal expansion) and can exclude low-quality images when integrated with a CADe system.

To evaluate CAQ stand-alone performance, the Endoscopic Image Quality test set, consisting of 647 images from 51 BE patients across 8 hospitals, was labeled for objective and subjective quality. To assess the benefit of the CAQ system as a preprocessing filter of a CADe system, the Barrett CADe test set was developed. It consisted of 956 video frames from 62 neoplastic patients and 557 frames from 35 non-dysplastic patients, in 12 Barrett referral centers.

**Results** As stand-alone tool, the CAQ system achieved Cohen’s Kappa scores of 0.73, 0.91, and 0.89 for objective quality, mucosal cleanliness, and esophageal expansion, comparable to inter-annotator scores of 0.73, 0.93, and 0.83. As preprocessing filter, the CAQ system improved CADe sensitivity from 82 % to 90 % and AUC from 87 % to 91 %, while maintaining specificity at 75 %.

**Conclusion** This study presents the first CAQ system for automated quality control in BE. The system effectively distinguishes poorly from well-visualized mucosa and enhances neoplasia detection when integrated with CADe.

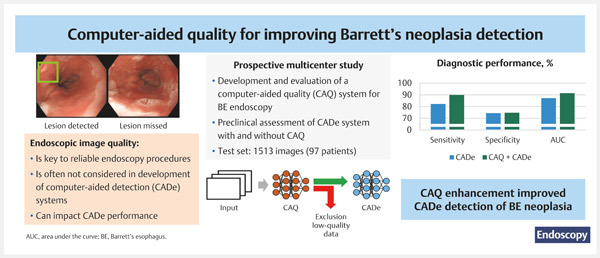

## Introduction

Timely detection of neoplasia in Barrett’s esophagus (BE) is critical but challenging, as early lesions often manifest with only subtle changes in mucosal and vascular patterns. Modern endoscopy systems allow visualization of nearly all such alterations. The ability of the endoscopist to recognize early lesions has thereby become the rate-limiting factor for early detection. Studies suggest that a substantial number of neoplastic lesions may be missed during BE screening and surveillance endoscopies 1 2 3 .

In response, several computer-aided detection (CADe) systems have been developed to assist endoscopists in recognizing Barrett’s neoplasia 4 5 6 7 8 . Although results have been promising, the effectiveness of these CADe systems is heavily dependent on the quality of the endoscopic procedure. This is for two reasons. First, CADe systems are often trained and tested solely with high-quality data from expert centers, making them vulnerable to heterogeneous data from daily clinical practice (i.e. domain shift) 9 . Second, similarly to human evaluation, if lesions are not properly visualized, they can also not be detected by CADe systems. Thorough esophageal cleansing, adequate inspection time, and complete mucosal visualization remain essential elements of a successful endoscopic examination 10 .

To improve endoscopic image quality and procedural standards during endoscopy, several computer-aided quality (CAQ) systems have been proposed 11 12 13 14 15 . These applications can monitor withdrawal speed, identify anatomic landmarks, or generate automated performance reports. For upper gastrointestinal endoscopy, current CAQ systems are mainly focused on detecting blind spots during screening endoscopies for gastric cancer 12 16 17 . There remains a need for CAQ systems developed specifically for esophageal inspection, for Barrett’s surveillance endoscopy, and for esophageal squamous cell carcinoma screening.

In this article, we introduce a novel CAQ system designed for BE endoscopy. We describe its development, assess its stand-alone performance across a large test dataset, and evaluate its effectiveness in an ex vivo setting when used in conjunction with a previously published CADe system 8 .

## Methods

### Setting and study design

This prospective multicenter study was conducted within the BONS-AI Consortium (Barrett’s Oesophagus imaging for Artificial Intelligence), which consists of 15 international centers with a tertiary referral function for management of early Barrett’s neoplasia and the Technical University of Eindhoven. All data utilized in this study were collected between 2020 and 2024, either as part of previous studies 7 8 or following the same protocol as these studies. This protocol was registered with the Dutch Trial Register under the number NL8411.

### Study aim

The aim of the study was to develop and evaluate a CAQ system for BE endoscopy. The system is designed to provide real-time feedback to the endoscopist on whether the esophageal mucosa is adequately visualized for reliable inspection based on various image quality parameters such as mucosal cleaning, esophageal expansion, and image clarity. In addition, the system should be capable of operating in conjunction with a previously published CADe system 8 .

### Image quality parameters

**Fig. 1:**
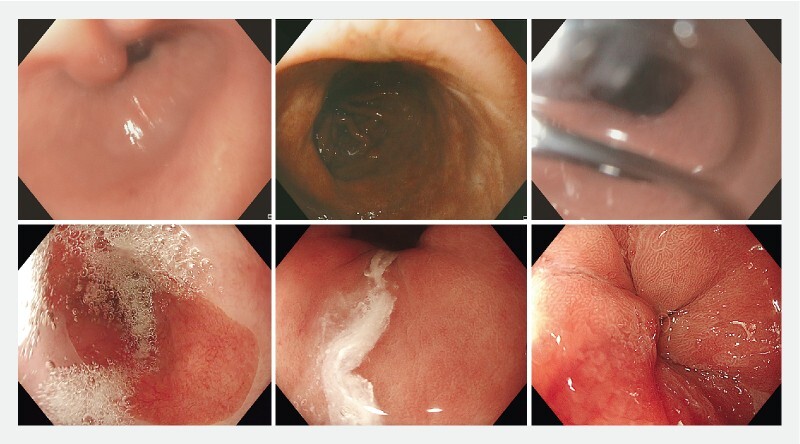
Examples of artifacts affecting objective (upper row) and subjective (lower row) image quality. Objective image quality is compromised by (from left to right): motion blur, poor illumination, and lens blur caused by bubbles. Subjective image quality is impaired by (from left to right): bubbles, mucus, and a collapsed esophagus.

We identified several key image quality parameters necessary for developing a CAQ system during a consensus meeting with three expert endoscopists. These parameters were categorized into two main types: objective image quality and subjective image quality. Objective image quality involves generic, quantifiable aspects of the image that directly influence its overall quality. Key criteria include factors such as illumination, various types of blur, and specular reflections. Subjective image quality is based on the context-specific characteristics of the image that require an endoscopist’s interpretation. Two critical factors were identified: a) esophageal cleaning – adequate mucosal cleaning is essential for thorough esophageal inspection, as the presence of mucus, bubbles, or debris can obstruct the view; b) esophageal expansion – for effective inspection, the esophagus needs to be sufficiently expanded, as a collapsed esophagus precludes proper visualization. Examples are given in Fig. 1 .

### Datasets

#### Pretraining

The CAQ system was first pretrained using the foundational dataset GastroNet-5 M. GastroNet-5 M comprises over 5 million unlabeled endoscopic images originating from throughout the gastrointestinal tract 18 . Previous studies by our consortium have shown that pretraining using GastroNet-5 M results in improved performance of endoscopic deep learning systems in terms of accuracy, robustness to data heterogeneity, and training efficiency 19 .

#### Training

After pretraining, the CAQ system underwent refinement training using prospectively collected endoscopic images and videos from patients with BE. All imagery was collected according to a standardized, multi-purpose acquisition protocol. For every 2-cm Barrett’s segment, including retroflexed position, a 10-second video was obtained with white-light endoscopy followed by two still images. This protocol has been described in more detail in a previous publication 7 . All imagery was obtained using Olympus HQ190 and EZ1500 endoscopes with CV190 and X1 processors (Olympus, Tokyo, Japan). MediCapture USB300 (MediCapture Inc., Plymouth Meeting, Pennsylvania, USA) and Sony HVO-4000MT (Sony, Tokyo, Japan) capture devices were used for image and video recording. After initial acquisition, specific images and video sequences were manually selected, aiming to represent the complete spectrum of endoscopic image quality. This included varying degrees of objective image quality (e. g. blurriness and illumination) and subjective image quality (i. e. esophageal cleaning and esophageal expansion). The final training set comprised 7463 images and video frames from 359 patients with nondysplastic and neoplastic BE, originating from 13 international Barrett’s referral centers, all part of our BONS-AI Consortium. All data were labeled with classification markers for various quality parameters, which are further described in the Ground truth development section below.

#### Evaluation

The CAQ system was evaluated using two independent test sets. First, to assess the CAQ system as a stand-alone system for image quality assessment during BE endoscopy, the endoscopic image quality test set was constructed. This test set originated from 8 of our 13 participating centers, all of which used the same protocol and the same equipment. The endoscopic image quality test set consisted of 647 images and video frames from 51 patients with nondysplastic BE. None of these patients were included in the training dataset. A total of 345 images were labeled for objective image quality and esophageal expansion, and 302 images were labeled for mucosal cleaning. Second, to evaluate the impact of the CAQ system on detection performance of a previously published CADe system 8 , the Barrett’s CADe test set was developed. The Barrett’s CADe test set consisted of 956 video frames from 62 patients with neoplastic BE and 557 video frames from 35 patients with nondysplastic BE, collected in 12 of our 13 participating centers. Video frames were specifically selected to comprise a wide variety of endoscopic image quality. This test set was used to assess whether applying the CAQ system as a filter to exclude low-quality images would improve the performance of the CADe system.

Datasets are summarized in Table 1 . Example cases of both test sets are displayed in **Fig. 1 s** in the online-only Supplementary material.

**Table 1:** Training and test datasets used for development and evaluation of the computer-aided quality system.

Dataset	Images/video frames (patients)
GastroNet-5 M pretraining dataset	5 084 494 (NA)
CAQ training set	
Objective image quality	4926 (243)
Subjective image quality – esophageal expansion	4025 (239)
Subjective image quality – mucosal cleaning	2537 (179)
Endoscopic image quality test set	
Objective image quality	345 (25)
Subjective image quality – esophageal expansion	345 (25)
Subjective image quality – mucosal cleaning	302 (26)
Barrett CADe test set	
Early Barrett’s neoplasia	956 (62)
Nondysplastic Barrett’s esophagus	557 (35)

CADe, computer-aided detection; CAQ, computer-aided quality; NA, not applicable.

### Ground truth development

**Fig. 2:**
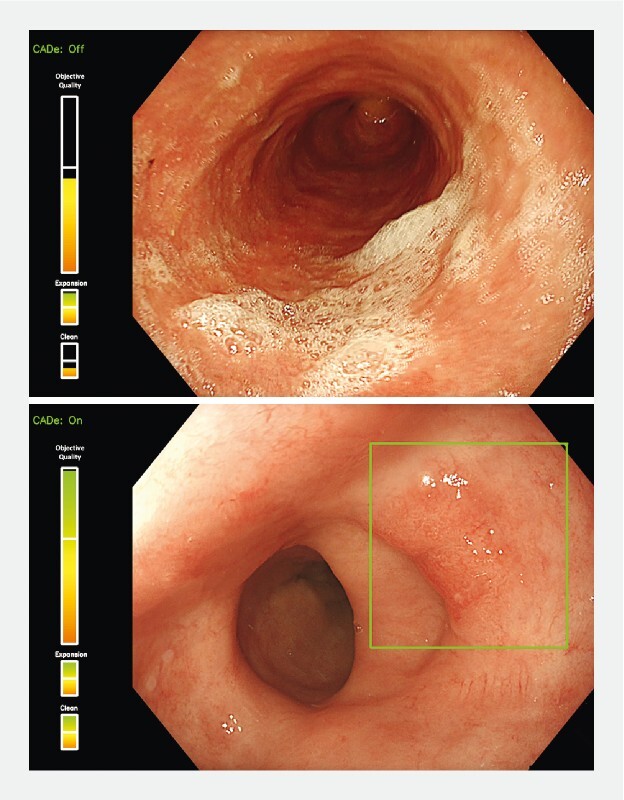
Graphical user interface of the integrated computer-aided quality (CAQ) + computer-aided detection (CADe) system. The upper bar displays the objective image quality assessment, while the two lower bars indicate subjective image quality metrics (i. e. esophageal expansion and mucosal cleanliness). The CADe system is activated only when all quality parameters meet the required standards.

All image quality parameters described above were used as ground truth for manual data annotation of training data and the endoscopic image quality test set. Objective image quality was rated on a scale from 1 to 5, with 1 indicating minimal identifiable mucosa due to quality artifacts and 5 representing a clear image with perfectly visible mucosa. Esophageal expansion was categorized as either “open” or “collapsed.” Esophageal cleaning was assessed on a scale of “inadequate,” “satisfactory,” and “optimal,” reflecting the mucosal cleanliness of the esophagus. These categories align loosely with the recently published Gastroscope RAte of Cleanliness Evaluation (GRACE) score 20 , where “inadequate” corresponds to extensive mucosal obstruction (severe presence of mucus, bubbles, biliary fluid, and/or foam covering more than 50 % of the surface), “satisfactory” indicates moderate obstruction (coverage between 5 % and 50 % of the surface, with visibility sufficient for evaluation), and “optimal” denotes minimal to no obstruction (coverage less than 5 % of the surface, allowing clear mucosal visibility). Examples of annotated images are illustrated in **Fig. 2 s** .

All training data were annotated by three research fellows (J.J., R.E.H., M.J.) under supervision of experts (A.G., J.B.), with each image reviewed by a single annotator. For the test datasets, each image and video frame was independently reviewed by two annotators.

For the Barrett’s CADe test set, ground truth criteria were similar to our previous studies 7 8 . First, a histologic gold standard was applied: images classified as neoplastic were derived from cases confirmed to have high grade dysplasia or adenocarcinoma in the corresponding endoscopic resection specimen, while nondysplastic BE cases were selected based on the absence of any dysplasia, as confirmed by the Seattle random biopsy protocol. Second, visual criteria (as assessed by two expert endoscopists) were adhered to: neoplastic cases were required to display a visible lesion suspicious for neoplasia, whereas nondysplastic BE cases were limited to flat BE segments without any suspicious abnormalities.

### Model development

The CAQ system was developed to address three key tasks: the assessment of objective image quality, esophageal expansion, and esophageal cleaning. For simplicity, we trained separate networks for each task. All models utilized a GastroNet-5M-pretrained ResNet-50 architecture as encoder for feature extraction. Further details regarding architectural design choices, model training, and optimization are provided in the Supplementary material.

During the inference phase, each image or video frame is processed sequentially through the three separate networks, with each network independently evaluating its designated image quality parameter. If the quality parameters meet the predefined standards – objective image quality scored > 3, esophageal expansion classified as “open,” and esophageal cleaning rated as at least “adequate” – the CAQ algorithm provides a positive signal. Otherwise, a negative signal is provided to the endoscopist.

### Integrated CAQ-CADe system

Our previously published CADe system was developed using a large multicenter training set and has been rigorously evaluated across multiple test sets and benchmarked by over 100 general and expert endoscopists 8 . However, as CADe systems require a certain level of endoscopic image quality to function effectively, we integrated the newly developed CAQ system into the CADe algorithm as a preprocessing filter. In this framework, images or video frames that do not meet the predefined image quality standards are excluded from further CADe analysis ( **Fig. 3 s** ). The CAQ system thus ensures that the CADe system operates under ideal conditions, thereby reducing the likelihood of missed lesions or false-positive alarms. The graphical user interface designed for this integrated CAQ-CADe system is illustrated in Fig. 2 .

### Outcome measures

#### Image quality assessment

The ability of the CAQ system to quantify endoscopic image quality was measured using the endoscopic image quality test set. Performance was evaluated by assessing agreement with annotator ratings across all image quality parameters (i. e. objective and subjective image quality).

#### Barrett’s neoplasia detection

For the Barrett’s CADe test set, both the stand-alone CADe system and the integrated CAQ-CADe system were evaluated for Barrett’s neoplasia detection. Classification was considered correct when the CADe system correctly classified an image as neoplastic or nondysplastic BE. Classification performance was displayed in terms of sensitivity, specificity, and area under the curve (AUC).

### Statistical analysis

We evaluated both the stand-alone performance of the CAQ system for image quality assessment and the impact of CAQ on detection rates of our CADe system. Given the absence of a perfect gold standard for endoscopic image quality, we employed agreement scores as our primary metric, using Cohen’s kappa for binary classifications (esophageal expansion) and weighted Cohen’s kappa for ordinal classifications (objective image quality and mucosal cleaning). For Barrett’s neoplasia detection, we compared sensitivity, specificity, and AUC scores between the stand-alone CADe system and the integrated CAQ-CADe system. Confidence intervals for sensitivity and specificity were calculated using Wilson’s method, and DeLong’s method was applied for AUC scores.

## Results

### Accuracy of stand-alone CAQ system

The CAQ system was evaluated in a stand-alone setting using the endoscopic image quality test set. For objective image quality assessment, the CAQ system achieved an average agreement score (Cohen’s kappa) of 0.73 with the annotators, which reflects substantial agreement. Agreement between the two annotators was similar, at 0.72. When evaluating subjective image quality, the CAQ system displayed agreement scores of 0.91 and 0.89 for assessment of the esophageal cleaning and expansion status, respectively. Both indicate near perfect agreement. Agreement between annotators was 0.93 and 0.83, respectively. Results are summarized in Table 2 .

**Table 2:** Agreement between the computer-aided quality system and human annotators, and between annotators.

Test set and subset	Mean CAQ–annotator agreement, Cohen’s κ (95 %CI)	Inter-annotator agreement, Cohen’s κ (95 %CI)
Endoscopic image quality		
Objective	0.73 (0.66–0.80)	0.72 (0.65–0.79)
Subjective – cleaning	0.91 (0.87–0.95)	0.93 (0.89–0.97)
Subjective – expansion	0.89 (0.84–0.94)	0.83 (0.77–0.89)

CAQ, computer-aided quality.

### Combined CAQ-CADe performance

The CAQ system was then evaluated as a preprocessing filter for the CADe system. For baseline CADe performance, CADe alone was evaluated using the Barrett’s CADe test set, achieving sensitivity, specificity, and AUC scores of 82.2 %, 74.7 %, and 87.2 %, respectively. Upon integrating the CAQ system into the CADe workflow, 640 /1513 images were excluded based on endoscopic image quality (442 neoplastic images and 198 nondysplastic images). This resulted in substantially improved performance. Sensitivity and AUC increased to 89.9 % and 91.4 %, respectively, while specificity remained at 74.7 % (see Table 3 ).

**Table 3:** Performance of the integrated computer-aided quality computer-aided detection (CADe) system on the Barrett CADe test set.

	Performance metric			Excluded images	
	Sensitivity, % (95 %CI)	Specificity, % (95 %CI)	AUC, % (95 %CI)	Neoplastic	Nondysplastic
CADe	82.2 (79.7–84.5)	74.7 (70.9–78.1)	87.2 (79.9–94.5)	–	–
CAQ + CADe	89.9 (87.2–92.1)	74.7 (70.0–78.9)	91.4 (82.0–100)	442	198

AUC, area under the curve; CADe, computer-aided detection; CAQ, computer-aided quality.

More details on excluded data with corresponding CADe performance are given in **Table 1 s** . CADe performance stratified for image quality scores of the CAQ system is given in **Fig. 4 s** .

## Discussion

Currently, the majority of AI research in gastrointestinal endoscopy focuses on CADe and CADx systems. However, these systems often fail to increase the endoscopic yield of clinically relevant findings. A significant limitation of these systems is that they can only assess the mucosal areas that are adequately visualized by the endoscopist. Lesions that are poorly visualized or not seen at all cannot be detected, even with the assistance of CADe.

Another challenge of current CADe/x systems is that they are often developed in expert centers under optimal imaging conditions. As a result, their training data do not represent the heterogeneous image quality encountered in community centers. This introduces a so-called domain gap, which further limits the effectiveness of these systems.

Interestingly, one of the few AI systems to date that demonstrated a clinically relevant outcome involved a CAQ system, designed to keep track of withdrawal speed during colonoscopy 21 . This system presumably increased the total mucosal area that was adequately imaged. Another more recent study successfully combined quality control with lesion detection for early gastric cancer recognition 17 . Therefore, the focus of endoscopic AI research may shift toward improving endoscopic procedural quality and endoscopic image quality.

In this study, we developed and evaluated a preliminary CAQ system for BE endoscopy using white-light endoscopy in an ex vivo setting. When tested as a stand-alone system, the CAQ system achieved excellent performance scores across a large test set that included a wide variety of endoscopic image quality. When used as a preprocessing filter, the CAQ system substantially increased the detection rate of a CADe system for early Barrett’s neoplasia.

To assess the performance of the CAQ system, we used two distinct test sets. The endoscopic image quality test set comprised video frames that exhibited a wide range of endoscopic image quality. These frames were annotated for both objective image quality – evaluated based on factors such as image clarity, illumination, and reflections – and subjective image quality, which included esophageal cleaning and expansion. The agreement between the CAQ system and the human annotators was comparable to the inter-annotator agreement, demonstrating the system’s consistency with expert evaluation. Notably, agreement scores for objective quality assessment were lower compared with subjective quality assessment, for both inter-annotator and annotator–AI agreement. This discrepancy may be explained by the use of a more granular 5-point scale for objective image quality, as opposed to the simpler 2- or 3-point scales used for subjective quality metrics. Additionally, the subjective cleaning metric was loosely adapted from the GRACE criteria 20 , which provide a structured framework. In contrast, no standardized criteria currently exist for evaluating objective image quality parameters in endoscopy.

The Barrett’s CADe test set included 1513 video frames from 97 patients with either neoplastic or nondysplastic BE. When we applied the CAQ system to exclude frames of suboptimal quality, only frames with sufficient image quality were evaluated by the CADe system. This improved the reliability of the CADe system’s outputs by focusing only on adequately visualized mucosal areas. Sensitivity and AUC scores of the CADe system increased from 82 % and 87 %, respectively, to 90 % and 91 %.

In total, 442 neoplastic and 198 nondysplastic frames were excluded. Notably, only two patients had all their frames excluded due to low quality, while the remaining patients retained some frames in the test set. The majority of these excluded frames were filtered based on objective image quality issues, such as motion blur and poor illumination. Among the excluded images, the CADe system demonstrated substantial lower performance, with sensitivity, specificity, and AUC scores of 73 %, 75 %, and 82 %, respectively.

When further evaluating specific subgroups of excluded data ( **Table 1 s** ), a few key patterns emerged. Poor objective image quality and inadequate esophageal expansion were primarily associated with reduced detection rates by the CADe system, with sensitivity scores of 68 % and 64 %, respectively. This may be explained by the fact that images with poor objective quality or collapsed esophageal tissue provide limited information (e. g. blurry or poorly illuminated areas), making detection by CADe less likely. Meanwhile, inadequate cleaning resulted in more false positives, with a specificity of 61 %. Unlike blurred or poorly illuminated images, images with inadequately cleaned mucosa are much more information dense. These images may contain artifacts such as mucus, bubbles, and debris, which can prompt the CADe system to falsely predict neoplasia. This underlines the importance of a CAQ system working in conjunction with CADe. By providing feedback to the endoscopist on image quality and filtering low-quality data for CADe, the CAQ system may help minimize missed lesions by alerting the endoscopist to inadequate image quality, allowing for adjustments in real time. Additionally, it can reduce false-positive detection by filtering low-quality images that might otherwise mislead the CADe system into falsely predicting neoplasia.

While the exclusion of suboptimal images improved the performance of the CADe system in well-visualized areas, it also meant discarding 442 frames that contained neoplastic lesions. Excluding suboptimal imagery can potentially lead to missed lesions if the endoscopist does not re-evaluate the region. This underscores the importance of adequate real-time feedback to the endoscopist, ensuring re-evaluation of poorly visualized areas. This should result in a synergistic effect, with the CADe system alerting the endoscopist to suspicious lesions, while the CAQ component provides clear feedback when image quality falls below the threshold for reliable assessment.

This study has several unique features. It describes the development of the first CAQ system specifically designed for esophageal inspection in general and BE specifically. The CAQ system was trained and evaluated on a substantial part of the largest BE dataset to date. Second, the CAQ system was evaluated in conjunction with a previously published CADe system. Evaluating the joint performance is critical for understanding how these systems can work together to optimize their diagnostic accuracy.

This study also has several limitations. First, all data used for development of the CAQ system originated from expert centers. Although data were specifically collected to comprise a wide variety of image quality (e. g. image collection before esophageal cleaning), this may still have led to some degree of selection bias. Second, although significant progress has been made with the GRACE score 20 , there is currently no established scientific or guideline-supported consensus on what defines high image quality in upper gastrointestinal endoscopy. Therefore, we identified several key factors that determine endoscopic image quality for the development of the CAQ system. However, these parameters have not been clinically validated. Third, the current CAQ system is computationally expensive, as it relies on three separate deep learning-based models for its tasks. Traditional, more efficient computer vision methods could serve as alternatives for evaluating factors such as blur and illumination. However, the current design prioritizes the robustness of deep learning. To enable efficient real-time performance, the CAQ system will undergo further optimization in future studies. Fourth, the CAQ system has only been tested in an ex vivo setting, and clinical validation remains to be performed. Real-time clinical evaluation will also present new opportunities: it will allow real-time feedback of the CAQ system to the endoscopist. This interaction between AI and endoscopist may substantially enhance endoscopic image quality, which in turn could improve the diagnostic performance of both endoscopists and CADe systems. Finally, the current CAQ system provides feedback on a per-frame basis, making it highly suitable as a preprocessing filter for the CADe system. However, it is not yet capable of offering a reliable quality indicator score for the complete endoscopic procedure. Ideally, a CAQ system would be capable of identifying blind spots or mucosal areas that have not yet been properly visualized. Achieving this level of functionality is challenging as it requires a certain degree of spatial awareness of the system. This is particularly difficult in BE owing to the lack of distinct anatomic landmarks – especially in long segments without squamous islands – and the dynamic movement of the esophagus caused by peristalsis and insufflation. However, this study is a step toward a procedure-wide CAQ system.

In conclusion, we present the first CAQ system specifically designed for automated quality control in BE endoscopy. This system effectively distinguishes between poor and good mucosal visualization. Additionally, the system functions as a preprocessing filter for an established CADe system, thereby guaranteeing a consistent level of endoscopic input quality. In future studies, we aim to enhance this prototype CAQ system by enabling comprehensive, procedure-wide feedback, including the detection of blind spots. We will also explore further integration of the CAQ system into our existing CADe models.

## Supplementary Material

Supplementary material Zusatzmaterial
