# Supplementary material for: The development and ex vivo evaluation of a computer-aided quality control system for Barrett’s esophagus endoscopy
Source: Endoscopy. 2025 Mar 6;57(7):709–16. doi: 10.1055/a-2537-3510 (PMC12204732; doi:10.1055/a-2537-3510)

The development and ex vivo evaluation of a computer-aided quality control system for Barrett's esophagus endoscopy

Martijn R. Jong, Tim J. M. Jaspers, Rixta A. H. van Eijck van Heslinga, Jelmer B. Jukema, Carolus H. J. Kusters, Tim G. W. Boers, Roos E. Pouw, Lucas C. Duits, Peter H. N. de With, Fons van der Sommen, Albert Jeroen de Groof, Jacques J. G. H. M. Bergman on behalf of the BONS-AI Consortium*

TABLE OF CONTENT

- Members and collaborators of the BONS-AI Consortium
- CAQ algorithm development
- Supplementary tables and figures
- References

***BONS-AI Consortium**

The authors wish to thank the members and collaborators of the BONS-AI Consortium, as listed below in alphabetical order:

Alaa Alkhalaf, Isala Hospital, Zwolle, the Netherlands;

Lorenza Alvarez Herrero, St. Antonius Hospital, Utrecht, the Netherlands;

Francisco Baldaque-Silva, Karolinska University Hospital, Stockholm, Sweden;

Maximilien Barret, Cochin Hospital, Paris, France;

Jacques J Bergman, Amsterdam UMC, Amsterdam, the Netherlands;

Torsten Beyna, Evangelisches Krankenhaus Düsseldorf, Düsseldorf, Germany;

Tim G Boers, TU Eindhoven, Eindhoven, the Netherlands;

Lucas C Duits, Amsterdam UMC, Amsterdam, the Netherlands;

Rixta A H, Amsterdam UMC, Amsterdam, the Netherlands;

Peter Elbe, Karolinska University Hospital & Karolinska Institute, Stockholm, Sweden;

Albert J de Groof, Amsterdam UMC, Amsterdam, the Netherlands;

Martin H Houben, HagaZiekenhuis, The Hague, the Netherlands;

Martijn R Jong, Amsterdam UMC, Amsterdam, the Netherlands;

Tim J M Jaspers, TU Eindhoven, Eindhoven, the Netherlands;

Jelmer B Jukema, Amsterdam UMC, Amsterdam the Netherlands;

Carolus H. J. Kusters, TU Eindhoven, Eindhoven, the Netherlands;

Rosalie C Mallant-Hent, Flevoziekenhuis, Almere, the Netherlands;

Guiomar Moral Villarejo, Nottingham University Hospitals NHS Trust, Nottingham, United Kingdom

Wouter Nagengast, UMC Groningen, Groningen, the Netherlands;

Jacobo Ortiz Fernández-Sordo, Nottingham University Hospitals NHS Trust, Nottingham, United Kingdom

Oliver Pech, St. John of God Hospital, Regensburg, Germany;

Roos E Pouw, Amsterdam UMC, Amsterdam, the Netherlands;

Krish Ragunath, Royal Perth Hospital, Perth, Australia;

Pieter Scholten, Onze Lieve Vrouwe Gasthuis, Amsterdam, the Netherlands;

Stefan Seewald, Klinik Hirslanden, Zurich, Switzerland;

Fons van der Sommen, TU Eindhoven, Eindhoven, the Netherlands;

Jessie Westerhof, UMC Groningen, Groningen, the Netherlands;

Bas L Weusten, UMC Utrecht & St. Antonius Hospital, Utrecht, the Netherlands;

Peter H de With, TU Eindhoven, Eindhoven, the Netherlands;

Development of CAQ system

The model development process began with pretraining on the GastroNet-5M dataset, which comprises over 5 million unlabeled endoscopic images spanning the entire gastrointestinal tract[1]. As the pretraining objective, we employed the "self-distillation with no labels" (DINO) method.[2] The ResNet-50 encoder was pretrained for 200 epochs, adhering closely to the hyperparameters outlined in the original DINO publication.

Following pretraining, the ResNet-50 encoder served as a feature extractor for three key tasks. The encoder was kept frozen, and a distinct classification head was trained for each task in separate training cycles. Each classification head consisted of a single MLP layer directly connected to the ResNet-50 encoder. Training for each head was conducted over 50 epochs, with the model checkpoint exhibiting the lowest validation loss retained.

To evaluate objective image quality, mean squared error (MSE) loss was employed to quantify the difference between predicted and ground truth labels. For the other two tasks—esophageal expansion and esophageal cleaning—binary cross-entropy loss was applied to optimize classification performance. All images were resized to a standardized resolution of 256×256 pixels, and training was conducted with a batch size of 128 images. Data augmentation was minimal, incorporating only random translations, horizontal and vertical flips, and 90-degree rotations.

REFERENCES

1. Boers TGW, Fockens KN, van der Putten JA, Jaspers TJM, Kusters CHJ, Jukema JB, et al. Foundation models in gastrointestinal endoscopic AI: Impact of architecture, pre-training approach and data efficiency. Medical Image Analysis. 2024:103298.
2. Caron M, Touvron H, Misra I, Jégou H, Mairal J, Bojanowski P, et al. Emerging Properties in Self-Supervised Vision Transformers2021 April 01, 2021:[arXiv:2104.14294 p.]. Available from: https://ui.adsabs.harvard.edu/abs/2021arXiv210414294C.

Table 1s Numbers of excluded images and corresponding CADe performance. NDBE = nondysplastic Barrett's esophagus. OIQ = objective image quality.

Data	# images		CADe performance		
	Neoplasia	NDBE	Sensitivity (95% CI)	Specificity (95% CI)	AUC (95% CI)
**All excluded images**	442	198	73.3% (69.0% - 77.2%)	74.8% (68.3% - 80.3%)	82.4% (70.8% - 94.0%)
- **OIQ <3**	346	182	67.6% (62.5% - 72.3%)	75.8% (69.1% - 81.4%)	79.5% (67.8% - 91.2%)
- **Esophagus collapsed**	193	106	63.7% (56.7% - 70.2%)	80.2% (71.6% - 86.7%)	82.7% (66.8% - 98.6%)
- **Inadequate cleaning**	165	31	78.8 (71.9% - 84.3%)	61.3% (43.8% - 76.3%)	82.1% (53.1% - 100%)

Fig. 1s Example cases of the endoscopic image quality test set (rows 1 & 2) and the Barrett CADe test set (rows 3 & 4).

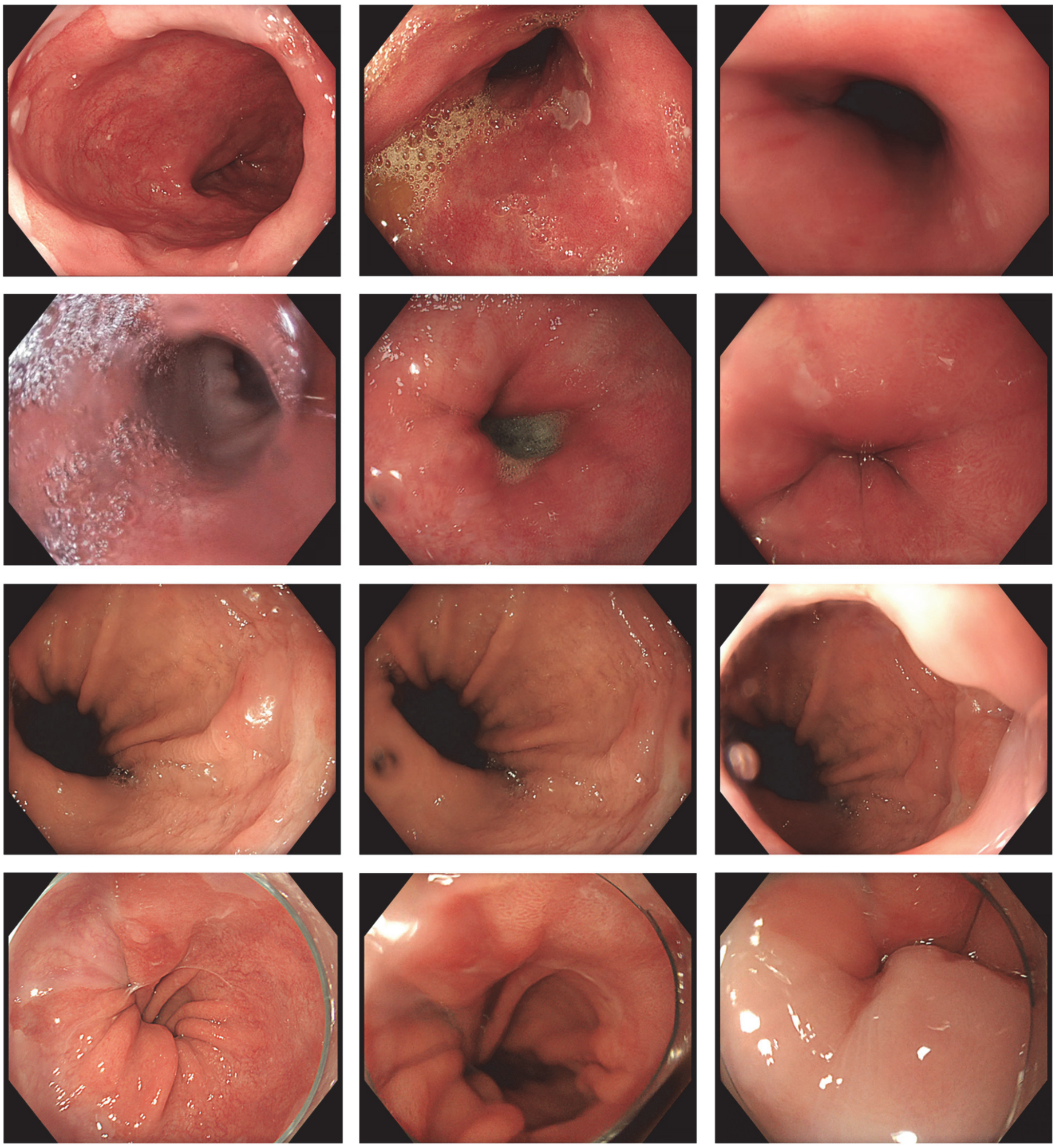

Fig. 2s Example images with annotations according to our proposed endoscopic image quality framework for Barrett's esophagus endoscopy.

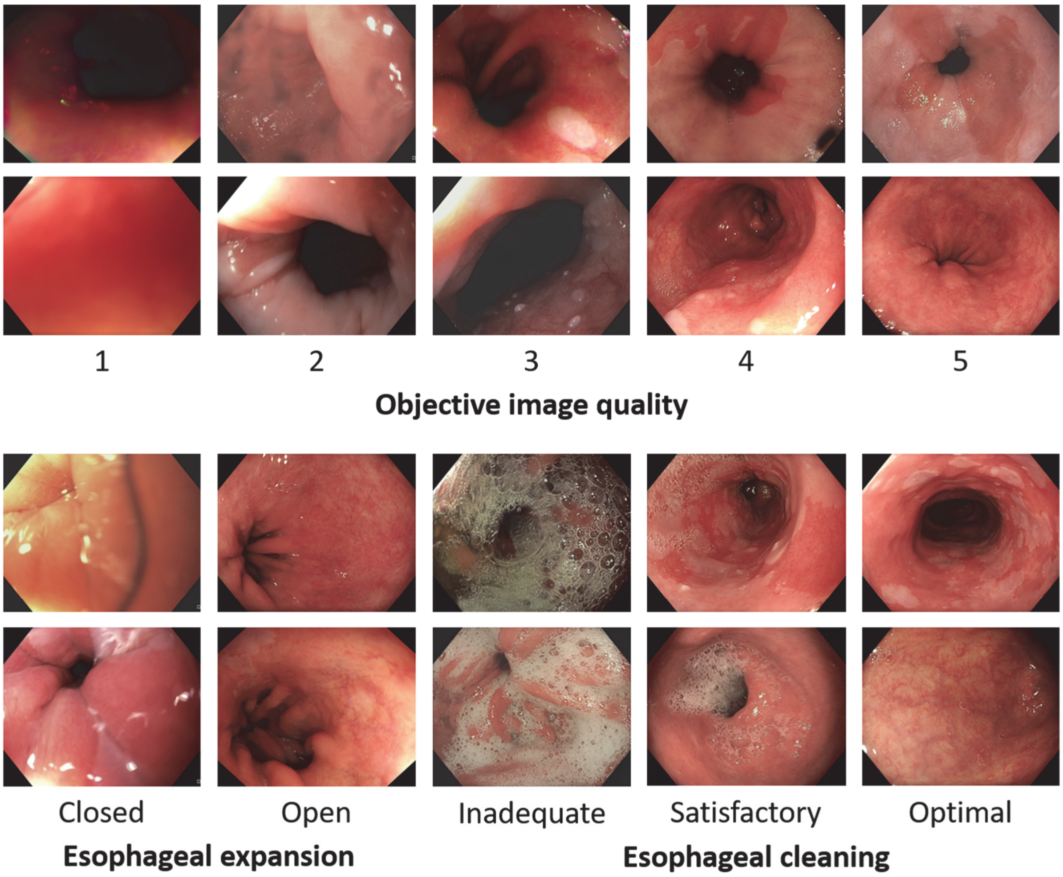

Thieme

Fig. 3s Workflow in CAQ-CADe integration. During inference, input images are passed, in parallel, through the three networks of the CAQ system. If the image complies with all predefined image quality standards, the image is forwarded through the CADe system. Otherwise, the image is excluded. OIQ, objective image quality.

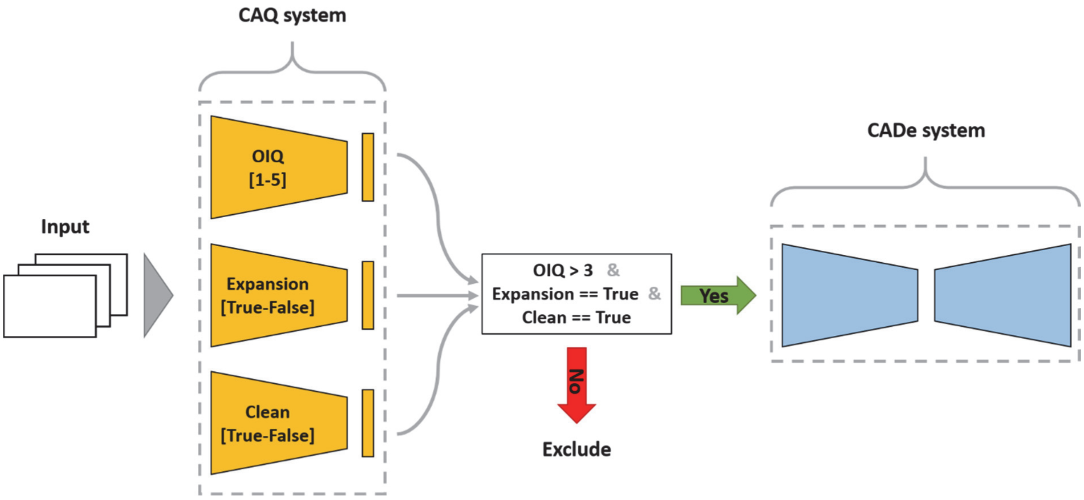

Fig. 4s Distribution of CADe neoplasia scores for various CAQ objective image quality (OIQ) scores.

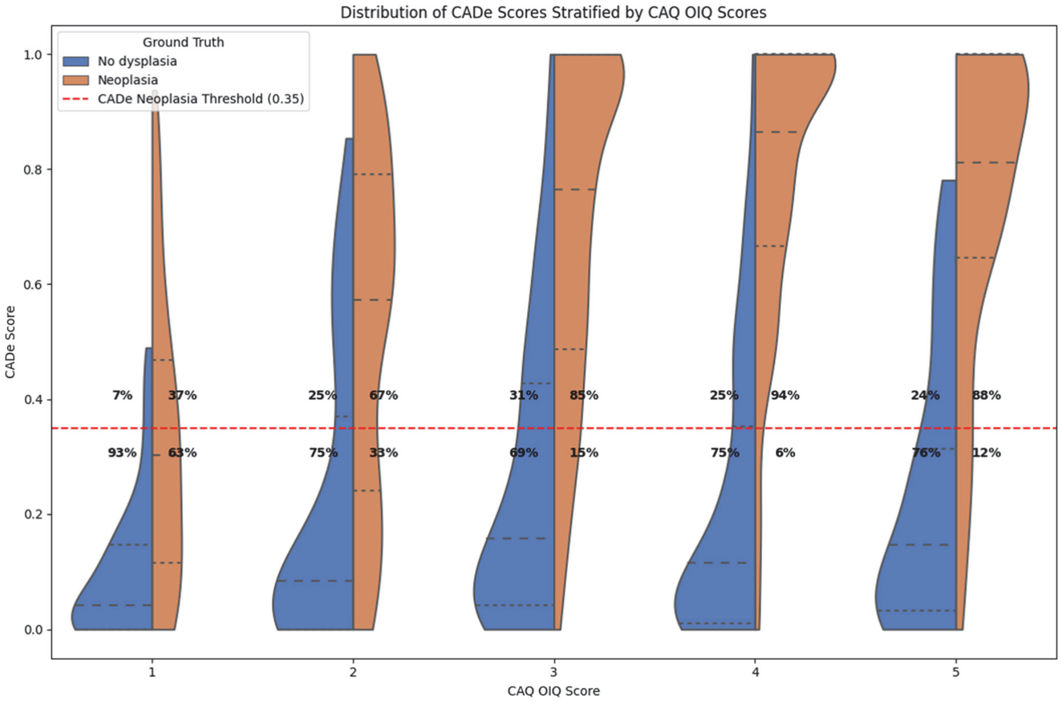

Supplement: Supplementary file 1 — Supplementary material [file 24857supmat_10-1055-a-2537-3510.pdf]
